# Comparison of Outcomes between Early Fascial Closure and Delayed Abdominal Closure in Patients with Open Abdomen: A Systematic Review and Meta-Analysis

**DOI:** 10.1155/2014/784056

**Published:** 2014-06-02

**Authors:** Yu Chen, Jinning Ye, Wu Song, Jianhui Chen, Yujie Yuan, Jianan Ren

**Affiliations:** ^1^Department of Surgery, Jinling Hospital, Medical School of Nanjing University, Nanjing, Jiangsu 210002, China; ^2^Minimally Invasive Department of General Surgery, Fushun Central Hospital, Fushun, Liaoning 113006, China; ^3^Department of Gastrointestinal-Pancreatic Surgery, The First Affiliated Hospital of Sun Yat-sen University, 58 2nd Zhongshan Road, Guangzhou, Guangdong 510080, China

## Abstract

Up to the present, the optimal time to close an open abdomen remains controversial. This study was designed to evaluate whether early fascial abdominal closure had advantages over delayed approach for open abdomen populations. Medline, Embase, and Cochrane Library were searched until April 2013. Search terms included “open abdomen,” “abdominal compartment syndrome,” “laparostomy,” “celiotomy,” “abdominal closure,” “primary,” “delayed,” “permanent,” “fascial closure,” and “definitive closure.” Open abdomen was defined as “fail to close abdominal fascia after a laparotomy.” Mortality, complications, and length of stay were compared between early and delayed fascial closure. In total, 3125 patients were included for final analysis, and 1942 (62%) patients successfully achieved early fascial closure. Vacuum assisted fascial closure had no impact on pooled fascial closure rate. Compared with delayed abdominal closure, early fascial closure significantly reduced mortality (12.3% versus 24.8%, RR, 0.53, *P* < 0.0001) and complication incidence (RR, 0.68, *P* < 0.0001). The mean interval from open abdomen to definitive closure ranged from 2.2 to 14.6 days in early fascial closure groups, but from 32.5 to 300 days in delayed closure groups. This study confirmed clinical advantages of early fascial closure over delayed approach in treatment of patients with open abdomen.

## 1. Introduction

Nowadays, an open abdomen, defined as a laparotomy that is completed without closing abdominal fascia or skin intentionally, is widely performed in patients with severe sepsis or trauma. However, the unclosed abdomen is often a nightmare for surgeons and causes a heavy burden to public health resources in some local communities. A temporary abdominal closure (TAC), which is generally performed after an open abdomen, is indispensable to reduce the incidence of enteroatmospheric fistula or other complications. Up to the present, numerous TAC techniques have been described and applied into clinical practice, with improved outcomes realized [1–6]. The ultimate goal of TAC procedure is to achieve definitive fascial closure [7, 8].

Generally, this permanent closure could be performed early or late after a TAC procedure [9]. Early fascial closure is defined as a reapproximated closure of abdominal fascia within the window of 2-3 weeks after an open abdomen, whereas delayed abdominal closure, administrated with absorbable or nonabsorbable synthetic grafts as well as organic meshes [1, 10], is an alternative reconstructive operation for the unclosed abdomen. This closure is typically completed 6–12 months or longer after an open abdomen [11]. To improve survival rate and hospital service utilization, early fascial closure is routinely preferred to achieve a permanent abdominal closure. Meanwhile, this traditional viewpoint has brought great challenges to the surgical management of patients with open abdomen [12].

For the past 30 years, numerous techniques have been introduced to achieve a higher rate of early fascial closure after an open abdomen. Nevertheless, early fascial closure may not be feasible or prudent for specific patients with critical illness [13]. A forced fascial closure in early stage of open abdomen may lead to intra-abdominal hypertension (IAH), which is related to subsequent multiple organ dysfunction syndrome (MODS) and additional laparotomies. Besides, early fascial closure for patients with extensive abdominal wall defects would result in at least 50% recurrence rate of abdominal wound dehiscence [14].

It has been noticed that early fascial closure may be associated with a high mortality rate of open abdomen due to its induced visceral compression and IAH [15]. By contrast, delayed abdominal closure with planned surgical procedures (retention sutures, permanent or absorbable prosthetic mesh implantation, towel clip skin closure, zipper closure, etc.) would effectively prevent the occurrence of iatrogenic hypertension [16]. Although the delayed closure often leads to a planned ventral hernia, it earns growing popularity in specific conditions compared with early fascial closure [17].

The optimal way to achieve definitive abdominal closure for patients with open abdomen remains controversial. Surgeons are in a dilemma in making a choice between early fascial closure and delayed theme. Since various TAC methods have few impacts on permanent abdominal wall reconstruction [18], it is possibly reasonable to compare clinical outcomes of these two abdominal closure themes in open abdomen management.

Up to the present, comparative studies on clinical effects of different fascial closure methods for patients with open abdomen are limited, without randomized, controlled trials being reported yet. Hence, we systemically reviewed related observational trials on outcomes of fascial abdominal closure to further explore its role in open abdomen treatment.

## 2. Methods

### 2.1. Literature Search

We conducted an electronic bibliographic search in Medline, Embase, Cinahl, and Cochrane Library for studies from January 1950 to April 2013 to get all articles related to open abdomen treatment. The terms “open abdomen,” “laparotomy”, “open peritoneal cavity,” “celiotomy,” “abdominal closure,” “abdominal compartment syndrome,” “primary,” “delayed,” “permanent,” “fascial closure,” and “definitive closure” were used during the literature retrieving. In addition, personal files and relevant review articles in original articles were manually searched for additional studies, except journals and conference proceedings. Unpublished data were requested from trial authors by letters or mails when necessary. The search was not restricted to any language; however, only studies published in English, German, Spanish, or Dutch were included for final analysis.

### 2.2. Study Selection Criteria and Data Extraction

The criteria for selected studies were listed as follows.

1. Study design: prospective, retrospective, case series, or observational cohort studies. Reviews, a series of less than ten patients, nonconsecutive inclusion period, or series with single definitive abdominal closure technique in study population, were excluded from this meta-analysis.
2. Population: patients who underwent an open abdomen and survived through initial fascial closure attempt were enrolled as primary study group, whereas patients, who survived through skin grafting or mesh closure first and then underwent fascial closure in final stage of abdominal wall reconstruction, were enrolled as control group. Those who died prior to definitive abdominal closure, either early or delayed fascial closure, should be excluded for final analysis.
3. Intervention: early fascial abdominal closure within 2-3 weeks after initial laparostomy or any forms of delayed abdominal closure was considered to achieve a definitive reconstruction of abdominal wall after an open abdomen.
4. Outcomes: primary outcomes were mortality rate, length of intensive care unit (ICU) stay, length of total hospital stay, time to definitive abdominal closure, and incidence of postoperative complications. Mortality was defined as any death during hospitalization or within 30 days after a successful fascial closure. Postoperative complications should include intestinal fistula, intra-abdominal abscess, recurrent hernia, and wound complications. Early fascial closure rate was included to indicate the percentage of early closure in open abdomen management. Secondary outcomes were abdominal wall defect areas, health care costs, and duration of nutritional support when available.

We elected to include all relevant trials in this systematic review. Two qualified searchers (JNY, YC) independently extracted data from original studies by using a preformatted datasheet. The inclusion period, number of patients, age, gender, Injury Severity Score (ISS) and Acute Physical and Chronic Health Evaluation II (APACHE II) score, and TAC procedures were recorded. Final enrolled studies were confirmed by the two investigators together after a critical review in depth. The authors contacted corresponding authors or first authors of selected articles in case some of data were unclear. Some missing data failed to return due to time reason or inefficient IRB approval. All data extracted from enrolled studies were output to Review Manager (version 5.2, Cochrane Collaboration software), following the recommendation of the reporting of meta-analysis in PRISMA statement [19]. Data from each enrolled study were artificially divided into primary closure group and control group according to our review protocol. We defined critically ill patients as those who suffered from severe trauma injuries or severe abdominal disease.

### 2.3. Analysis and Data Derivation

Meta-analyses were planned to examine pooled estimate of overall mortality and any postoperative complications. In certain cases, values required for analysis could be estimated by calculation using reported results if they were neither reported in original articles nor obtained from data request communication. Random-effects meta-analyses of pooled estimates and risk ratios were performed by using Review Manager, which used inverse-variance weighting to calculate random-effects pooled summary estimates; confidence limits; a test for differences between study effects; and an estimate of between-study variance. The random-effects model allowed for heterogeneity between/within studies, and it was used in all meta-analyses, with confirmation through heterogeneity *χ* ^2^ and *I* ^2^ statistics. To investigate the source of heterogeneity in an attempt to reduce it, cohorts were divided into subgroups. Possible covariates were also examined as sources of heterogeneity. Data were combined to estimate the common relative risk (RR) of mortality and postoperative complications and to calculate the associated 95% confidence intervals (CIs). Some outcomes were not analyzed but presented in a descriptive way. All *P* values below 0.05 were considered statistically significant.

## 3. Results

### 3.1. Included Studies

The searches revealed 1897 articles. Based on the title, 357 articles remained. After reviewing abstracts, 162 articles were excluded because they did not meet the inclusion criteria. We identified 195 relevant abstracts and obtained complete articles. Of these, another 163 articles did not meet the inclusion criteria. The remaining 32 articles were included in this systematic review, with 33 case series available. These enrolled studies were performed between 1995 and 2013, with no randomized controlled trials found. Of note, two matched-pair studies were included, with one prospective design performed [20, 21]. The inclusion periods ranged from 18 to 167 (median, 60) months.

### 3.2. Patients

Twelve series described traumatic patients only [1, 8, 9, 20, 22–29], and additional 17 series included traumatic and nontraumatic patients [6, 18, 29–43]. Only four series described nontraumatic patients [21, 44–46]. In all, 3125 patients were included for the final analysis. The sex distribution was described in 28 series (85%), with the percentage of the male ranging from 40 to 83%. The mean age of enrolled subjects ranged from 32 to 47 years in 28 series (85%). The ISS was recorded in 20 series (61%), ranging from 19 to 35. Only three series (9%) recorded the APACHE II score (range of mean value, 17–26).

### 3.3. Early Fascial Closure Rate

For comparative purpose, studies focusing on early fascial closure or delayed abdominal closure alone were excluded from this review. The early fascial closure rate ranged from 29% to 85% (Table 1). In sum, 1942 (62%) patients achieved early fascial closure after a successful TAC procedure. Vacuum assisted fascial closure was described in 28 series (85%); however, this technique did not influence the weighted pooled fascial closure rate (72 versus 69%; *P* = 0.212; *I* ^2^ = 81%). The mean frequency of operations to achieve early fascial closure was 3.2, ranging from 2.2 to 8.8.

### 3.4. Primary Outcomes

#### 3.4.1. Mortality after a Definitive Closure

**Figure 1:**
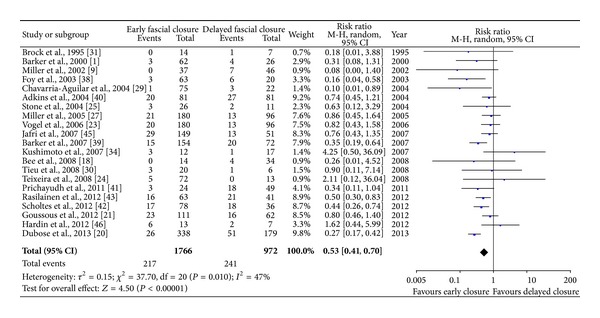
Early fascial closure versus delayed abdominal closure for mortality rate.

In this review, patients who died prior to a final abdominal closure were excluded from the calculation of mortality rate. Mortality was reported in 21 series (64%). The weighted pooled mortality rate was 12.3% in primary fascial closure group, compared with 24.8% in the control group. After excluding several studies with profound heterogeneity, the estimated mortality (random-effects model, Figure 1) indicated that early fascial closure had better effect than delayed approach in reducing the risk of mortality (risk ratio, 0.53; 95% CI 0.41–0.70; *P* < 0.0001). Sensitivity analysis indicated that published bias was not significant (*χ* ^2^ = 20.86; *P* = 0.110; *I* ^2^ = 33%). However, the period of follow-up was covered with a great distribution, ranging from 0.5 to 3.0 years.

#### 3.4.2. Complications

**Figure 2:**
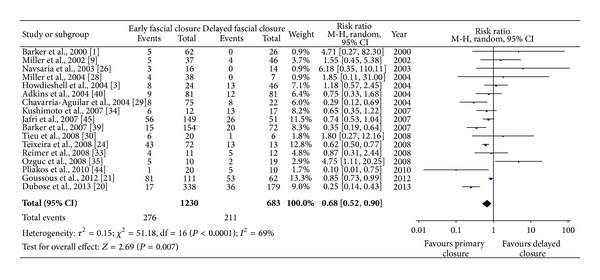
Comparison of postoperative complications after definitive closure between early fascial closure and delayed abdominal closure.

Postoperative complications, including wound complications, secondary fistula, recurrent hernia, and intra-abdominal abscess, were reported in 29 series (88%), most commonly for fistula (79%) and abscess (61%). By pooled analysis with random-effects model, the RR was 0.68, 95% CI (0.52–0.90), with low heterogeneity between selected studies (*P* < 0.0001, *I* ^2^ = 69%). Subgroup analysis, including fistula, abscess, wound infection, and hernia, revealed less incidence rate in early fascial closure populations. The weighted data were suggestive of a reduced risk of postoperative complications with early fascial closure after a TAC procedure (Figure 2).

#### 3.4.3. Time to Definitive Abdominal Closure

Time to definitive closure was reported in 28 (85%) of 33 case series. The mean duration to a definitive abdominal closure ranged from 2.2 to 14.6 days in early fascial closure groups, but from 32.5 to 300 days in the delayed closure groups. In the delayed closure populations, planned ventral hernia repair was performed in about 398 patients (34%).

#### 3.4.4. ICU Stay and Hospital Stay

**Figure 3:**
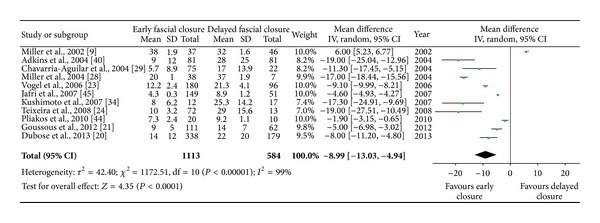
The mean length of ICU stay in early fascial or delayed closure populations. Estimated SD values in some trials were calculated from five percent of correlated mean values.

**Figure 4:**
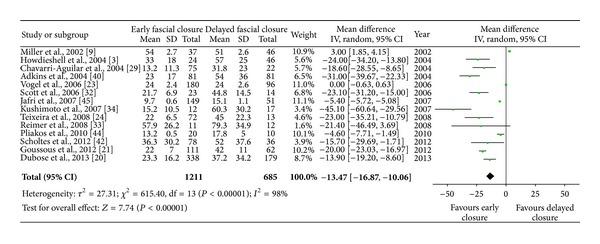
The mean length of total hospital stay for patients with early fascial closure or delayed closure. Estimated SD values in some trials were calculated from five percent of correlated mean values.

ICU stay was described in 16 series, with 21 series for total hospital stay. The mean length of ICU stay ranged from 4 to 38 days in early fascial closure groups and from 9 to 37 days in the delayed closure groups. The pooled estimates from random-effects model indicated a reduction in duration of ICU stay for primary closure group, weighted mean difference −8.99 (95% CI, −13.03, −4.94). For the length of hospital stay, early fascial closure had a reduced duration compared with the delayed theme. The mean length of hospital stay ranged from 10 to 58 days in early fascial groups and from 15 to 79 days in control groups. The overall stay in ICU (Figure 3) or hospital (Figure 4) was significantly shortened in early fascial closure populations as compared with the delayed closure; however, heterogeneity between enrolled trials was still significant (*P* < 0.00001, *I* ^2^ = 98%).

### 3.5. Secondary Outcomes

The abdominal wall defect areas were evaluated in only one study [32]. Health care costs were compared in eight series [18, 23, 27, 30, 31, 35, 39, 45]. The length of nutritional support (enteral or parenteral nutrition) for patients with open abdomen was mentioned in two series [6, 39]. These outcomes cannot be compared between two different closure groups due to limited data.

### 3.6. Publication Bias

Publication bias (funnel plot) was analyzed for all outcomes. Because of some unpublished data, there was no clear evidence of asymmetry and publication bias for enrolled studies or any of reported outcomes.

## 4. Discussion

In this systematic review, all findings indicate that early fascial closure still earns great popularity in treatment of patients with open abdomen, whereas delayed closure is mostly regarded as a second-choice method after a successful TAC procedure. However, the benefits of early fascial closure to clinical outcomes are not outstanding in certain fields, particularly for postoperative complications and length of ICU stay. Comprehensive resolution and good judgment are quite indispensable in open abdomen management, no matter which abdominal closure method is selected in clinical practice.

The enrolled studies are commonly retrospective nonrandomized trials, with only one prospective design included. Due to ethics constraints, patients who are suitable for early fascial closure after an open abdomen must immediately undergo an aggressive attempt rather than a late abdominal closure. Besides, most studies mainly compared effects of different TAC techniques on the fascial closure rate, rather than outcomes of different definitive abdominal closure strategies after a TAC procedure. The pivotal problem of this analysis is that most enrolled studies suffered from considerable bias in both patient and treatment selection, without adequacy of allocation concealment. The articles infrequently recorded scoring systems that evaluate the severity of enrolled patients (e.g., ISS and APACHE II score). Hence, this review cannot evaluate the impact of the severity of open abdomen on clinical outcomes. Besides, several variables of interests, such as area of abdominal wall defect, cost of health care, and length of nutrition therapy, were recorded in a few studies, and the heterogeneity among selected studies for some variables was too evident to compare between two abdominal closure groups.

Importantly, some factors, such as operation time, pain control, nutritional support, antibiotics administration, and nursing care, might have impacts on clinical treatment endpoints of open abdomen but failed to be explored due to limited data. Furthermore, early fascial closure was defined variously in many trials, lacking unified standard. Most studies considered a completion of fascial closure within 2-3 weeks after initial open abdomen surgery as early closure [3, 25, 33, 34, 47]. In this review, early fascial closure rate from weighted data is 62% (range, 29%–85%). Several studies [48–50], which reported relatively higher early fascial closure rate, were excluded from this study due to no comparison with delayed abdominal closure. Actually, those studies definitely made great contributions to the management of open abdomen.

The reduction in mortality, ICU, or hospital stay with early fascial closure is not hard to explain. Patients encouraged to undergo early fascial closure are often intact from extensive bowel edema, massive tension of abdominal wall, pulmonary or hemodynamic deterioration with closure, poor nutritional status, or severe sepsis. Those patients, as compared with critically ill patients, could have more chances to have a fast recovery from abdominal wall reconstruction procedures. Additionally, according to previous reports, patients with fecal contamination/peritonitis, massive transfusion, multiple abdominal injuries, hypothermia, acidosis, or coagulopathy still get a chance of undergoing early fascial closure [12].

Although numerous TAC techniques have been introduced to achieve a higher early fascial closure rate, several studies have indicated that the rate might be a result neither of selective collection of patients or different TAC methods nor of severity of primary disease [18, 29]. From this viewpoint, we have not categorized TAC technique in this meta-analysis. Nevertheless, we do believe that various TAC techniques are associated with different clinical outcomes for patients with open abdomen.

Early fascial closure earns great popularity in open abdomen treatment; however, the frequency of hernia from that aggressive procedure is unacceptably high. Moreover, early closure with meshes is a very controversial issue since exogenous implants may increase the risk of extensive adhesions [35]. In many centers, if early fascial closure cannot be performed, the skin is closed alone first, with the iatrogenic hernia repaired later by various surgical procedures. This therapeutic strategy circumvents mesh-related complications. Moreover, patients receiving this treatment have to live with a planned hernia for a long period and eventually need a second operation.

In the current review, the frequency of ventral hernia complicated with early fascial closure is not high as expected. Early closure following vacuum-assisted closure can effectively decrease intra-abdominal adherence and wound complications. As for delayed closure, the planned hernia can be safely repaired once initial injuries are resolved, and the skin graft can be easily dissected from the underlying tissue [1]. However, those surgical procedures are commonly performed 3 to 6 months later after the acute illness has been controlled. The long-term waiting and great expenditure are great challenges for both patients and health resources. Those embarrassing reasons may explain the declined application of delayed closure in open abdomen management.

The most serious complication associated with open abdomen therapy is gastrointestinal fistula [51]. Other complications, such as wound infection, intra-abdominal abscess, and recurrent hernia, are commonly reported. In this review, fistulae and abscesses are the most consistently observed complications after definitive abdominal closure. However, the actual incidence of postoperative complications is unable to obtain from the current available data since most included studies focus on roles of different TAC methods in open abdomen treatment.

Under certain circumstances, such as damage control, planned relaparostomy, significant visceral edema, and retroperitoneal hematoma, early fascial closure either is not practicable or could cause fascial apposition with excessive tension [52, 53]. Several previous studies have shown that the reduction of time until definitive abdominal closure is essential because open abdomen therapy is associated with increased morbidity and mortality [13, 25, 30].

Early fascial closure can be precluded by many factors, including persistent visceral edema, uncontrolled intra-abdominal infection, ileus-associated enteral nutrition intolerance, and refeeding syndrome due to long-term use of parenteral nutrition [54]. Besides, sustained intracranial hypertension, hypoxemia secondary to adult respiratory distress syndrome, and inadequate surgical procedures may be possible reasons for a failed attempt of early closure [3]. To improve early fascial closure rate, overfluid resuscitation must be avoided, and judicious fluid management should be implemented not only on admission, but also throughout the whole course of open abdomen management [21].

## 5. Conclusions

The current review and meta-analysis may indicate that early fascial closure has great clinical advantages in reducing the mortality and incidence of complications as compared with delayed abdominal closure. Aggressive attempt at early fascial closure should be considered first in the management of open abdomen.

## Figures and Tables

**Table 1:** Early fascial closure rate of all enrolled studies.

Study	Patients (*n*)	Early closure (*n*)	Delayed closure (*n*)	Rate (%)
Adkins et al., 2004 [40]	162	81	81	50
Barker et al., 2000 [1]	88	62	26	70
Barker et al., 2007 [39]	226	154	72	68
Bee et al., 2008 [18]	48	14	34	29
Brock et al., 1995 [31]	21	14	7	67
Chavarria-Aguilar et al., 2004 [29]	97	75	22	77
Dubose et al., 2013 [20]	517	338	179	65
Foy et al., 2003 [38]	83	63	20	76
Goussous et al., 2012 [21]	173	111	62	64
Hardin et al., 2012 [46]	20	13	7	65
Howdieshell et al., 2004 [3]	70	24	46	34
Jafri et al., 2007 [45]	200	149	51	75
Kritayakirana et al., 2010 [37]	60	34	26	57
Kushimoto et al., 2007 [34]	29	12	17	41
López-Quintero et al., 2010 [36]	14	7	7	50
Miller et al., 2002 [9]	83	37	46	45
Miller et al., 2004 [28]	45	38	7	84
Navsaria et al., 2003 [26]	30	16	14	53
Ozguc et al., 2008 [35]	74	33	41	45
Pliakos et al., 2010 [44]	30	20	10	67
Prichayudh et al., 2011 [41]	73	24	49	33
Rasilainen et al., 2012 [43]	104	63	41	61
Reimer et al., 2008 [33]	23	11	12	48
Scholtes et al., 2012 [42]	114	78	36	68
Scott et al., 2006 [32]	37	23	14	62
Stone et al., 2004 [25]	37	26	11	70
Teixeira et al., 2008 [24]	85	72	13	85
Tieu et al., 2008 [30]	26	20	6	77
Tremblay et al., 2001 [6]	100	42	58	42
Vogel et al., 2006 [23]	276	180	96	65
Weinberg et al., 2008 [8]	159	100	59	63
Yeh et al., 1996 [22]	21	8	13	38
